# Identification of Groundwater Contamination in a Rapidly Urbanized Area on a Regional Scale: A New Approach of Multi-Hydrochemical Evidences

**DOI:** 10.3390/ijerph182212143

**Published:** 2021-11-19

**Authors:** Pan Bi, Lixin Pei, Guanxing Huang, Dongya Han, Jiangmin Song

**Affiliations:** 1 School of Water Resources and Environment, Hebei GEO University, Shijiazhuang 050031, China; bipan1983@126.com; 2 Hebei Province Collaborative Innovation Center for Sustainable Utilization of Water Resources and Optimization of Industrial Structure, Hebei GEO University, Shijiazhuang 050031, China; 3 Haikou Marine Geological Survey Center, China Geological Survey, Haikou 571100, China; 4 Institute of Hydrogeology and Environmental Geology, Chinese Academy of Geological Sciences, Shijiazhuang 050061, China; handongyaycn@126.com (D.H.); 15612773009@163.com (J.S.)

**Keywords:** groundwater contamination, multi-hydrochemical evidences, porous aquifers, fissured aquifers, urbanization

## Abstract

Efficient identification of groundwater contamination is a major issue in the context of groundwater use and protection. This study used a new approach of multi-hydrochemical indicators, including the Cl-Br mass ratio, the hydrochemical facies, and the concentrations of nitrate, phosphate, organic contaminants, and Pb in groundwater to identify groundwater contamination in the Pearl River Delta (PRD) where there is large scale urbanization. In addition, the main factors resulting in groundwater contamination in the PRD were also discussed by using socioeconomic data and principal component analysis. Approximately 60% of groundwater sites in the PRD were identified to be contaminated according to the above six indicators. Contaminated groundwaters commonly occur in porous and fissured aquifers but rarely in karst aquifers. Groundwater contamination in porous aquifers is positively correlated with the urbanization level. Similarly, in fissured aquifers, the proportions of contaminated groundwater in urbanized and peri-urban areas were approximately two times that in non-urbanized areas. Groundwater contamination in the PRD was mainly attributed to the infiltration of wastewater from township-village enterprises on a regional scale. In addition, livestock waste was also an important source of groundwater contamination in the PRD. Therefore, in the future, the supervision of the wastewater discharge of township-village enterprises and the waste discharge of livestock should be strengthened to protect against groundwater contamination in the PRD.

## 1. Introduction

Groundwater resources are important sources of industrial, agricultural, and domestic water, especially in urbanized areas where there is an ever-increasing demand for water [1]. Unfortunately, many human activities, such as urban expansion and agricultural activities, lead to the deterioration of groundwater quality primarily due to infiltration from domestic sewage, industrial wastewater, landfill leachate, and fertilizers used on agricultural lands [2]. Thus, the identification of anthropogenic contributions to groundwater contamination is a major issue in the context of the use and management of groundwater resources in urbanized areas.

The Pearl River Delta (PRD) is one of the most rapidly urbanized and industrialized areas in China. For example, the area occupied by construction land in the PRD in 2006 was more than two times that in 1988 [3]. In this case, groundwater resources become more important in the PRD because of the ever-increasing water demand with the population influx. However, groundwater contamination in the PRD occurs as a result of infiltration from domestic sewage, industrial wastewater, and so on [4,5]. Therefore, it is important to identify the groundwater contamination sites in favor of the uses and management of groundwater resources in the PRD.

Some chemical parameters or their ratios, such as NO_3_^−^, organic contaminants, and the ratio of chloride–bromide (Cl/Br), are often indicators used to identify groundwater contamination. For instance, concentrations of groundwater NO_3_^−^ > 50 mg/L or organic contaminants > 0.05 μg/L were used to identify contaminated groundwater samples before the assessment of natural background levels in Portugal and Italy [6]. The presence of Cl and Br ions in waters shows that the Cl/Br ratio is an effective indicator for identifying groundwater contamination because these two ions have different abundances in natural fluids [7]. Additionally, high levels of PO_4_^3−^ and Pb and the occurrence of NO_3_ facies in groundwater are also useful indicators to identify groundwater contamination in the PRD because high levels of PO_4_^3−^ and Pb in groundwater in the PRD originated from various anthropogenic activities [2,4] and no NO_3_ facies occurred in groundwater in the PRD before 1980 [8,9].

The objectives of this study are to identify groundwater contamination in the PRD semi-quantitatively by multi-hydrochemical evidences and to analyze the main factors resulting in groundwater contamination in the PRD. The results would be in favor of the uses and protection of groundwater resources in the PRD. Here, multi-hydrochemical evidences include the concentrations of NO_3_^−^, PO_4_^3−^, organic contaminants, and Pb, the Cl/Br mass ratios, and the NO_3_ facies. To the best of our knowledge, it is the first time this approach has been used to identify groundwater contamination on a regional scale by using a combination of these hydrochemical indicators.

## 2. Study Area

### 2.1. Geographical Conditions

**Figure 1:**
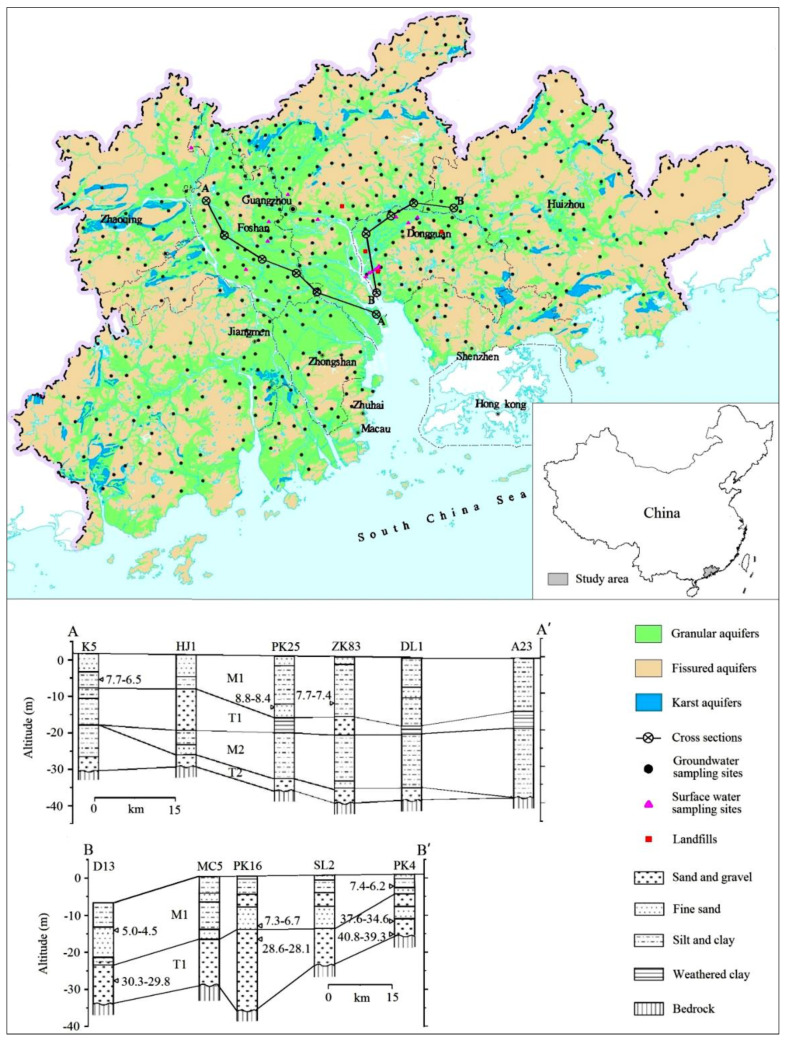
Hydrogeological setting and sampling sites in the Pearl River Delta.

As shown in Figure 1, the PRD is located within Guangdong province in southern China and covers a total area of 41,698 km^2^ with a population density of over 1500 inhabitants/km^2^ in 2018. It is bounded by hills in the east, west, and north and by the South China Sea in the south. The climate is typically subtropical monsoon, and the mean annual precipitation ranges from 1600 to 2300 mm/year. Three major rivers (e.g., East River, West River, and North River) merge in the south of the area and form the Pearl River, which finally discharges into the South China Sea.

### 2.2. Geological and Hydrogeological Settings

The PRD plain is located in the central and southern parts of the PRD and is covered by Quaternary sediments. Quaternary sediments consist of four stratigraphic sequences, including two marine sequences and two terrestrial sequences (Figure 1). The older marine sequence and the two terrestrial sequences were formed during the Pleistocene, while the younger marine sequence was formed during the Holocene. The older terrestrial sequence is the basal aquifer and is dominated by sand and gravel. By contrast, the younger terrestrial sequence is often a local aquifer that can be sandy fluvial deposits. These aquifers are porous and mainly recharged by vertical infiltration of precipitation, agricultural irrigation, and the lateral flow of rivers. Groundwater exploitation for domestic use is generally from the local aquifers but not the basal aquifers. In addition, porous aquifers in coastal areas are often intruded on by seawater [9]. The PRD plain is surrounded by hills in the east, west, and north, where fissured aquifers occur. The bedrocks include shale, sandstone, limestone, dolomite, granite, and gneiss, ranging in age from Cambrian to Tertiary, and crop out in these hilly areas. The karst aquifers account for less than 10% of the total area and are also around the PRD plain. The general direction of the regional groundwater flow in the aquifers of the PRD is northwest and northeast toward the coast [2].

### 2.3. Characteristics of Urbanization in the PRD

According to different urbanization levels, the land use in the PRD can be divided into three types, such as urbanized area (UA), peri-urban area (PUA), and non-urbanized area (NUA) [10]. The UA is associated with large-scale construction land and is characterized by dense populations, many factories, and huge productions of domestic sewage (Figure S1). The PUA is the epitaxial area (~2 km outside UA) of the UA and has fewer factories and population compared to UA [11]. The NUA mainly refers to woodlands, small villages, and agricultural lands [1]. In addition, landfills have been formed in the PUA and NUA during urbanization.

## 3. Materials and Methods

### 3.1. Sampling and Analytical Techniques

In 2006, a total of 399 groundwater samples were collected once from the porous aquifer (258 samples), fissured aquifer (132 samples), and karst aquifer (9 samples), respectively. In addition, 16 surface water samples (2 from drinking water source areas, 3 from estuaries, and 11 from rivers), and 4 landfill leachate samples were collected. All the samples were stored at 4 °C until the laboratory procedures could be performed. A multiparameter instrument (WTW Multi 340i/SET, Munich, Germany) was used to measure 3 on-site parameters, including redox potential (Eh), pH, and dissolved oxygen (DO). Inductively coupled plasma mass spectrometry (Agilent 7500ce ICP-MS, Tokyo, Japan) was used to measure metals including K^+^, Na^+^, Ca^2+^, Mg^2+^, and Pb in waters. Chemical oxygen demand (COD) was analyzed using a potassium dichromate titration method. Total dissolved solids (TDS) were measured using a gravimetric method. HCO_3_ was analyzed by an acid–base titration method. PO_4_ was measured by the molybdenum blue method. Ion chromatography (Shimadzu LC-10ADvp, Kyoto, Japan) was used to analyze NH_4_^+^ and other anions such as NO_3_^−^, SO_4_^2−^, Cl^−^, and Br^−^. To ensure data quality, each sample was analyzed in triplicate, and sample batches were regularly interspersed with standards and blanks. The relative errors of inorganic parameters were <±6%. The detection limits for the above inorganic parameters are in Table S1 (Supplementary Material). When the Br^−^ concentration was below the detection limit (0.1 mg/L), 0.09 mg/L was used as the Br^−^ concentration to calculate Cl/Br mass ratios. Gas chromatography–mass spectrometry (Agilent 5975 MS, Santa Clara, CA, USA) was used to measure 55 selected organic chemicals in this study. A list of these selected organic chemicals is shown in Table S2. The detection limits of these organic chemicals were 0.5–1 μg/L. Quality control of all analyzed organic chemicals followed the US EPA protocols, in which the recovery of surrogates was in a range of 83% to 115%.

### 3.2. Socioeconomic Data

Socioeconomic parameters in 2006 of various cities in the PRD were used in this study. Relevant data were obtained from the Statistical Yearbook of Guangdong Province [12], as shown in Table S3. Note that the differences in the socioeconomic parameters in various cities caused by the differences in the sizes of the cities were eliminated by using the values of socioeconomic parameter values per square kilometer in this study.

### 3.3. Hierarchical Cluster Analysis and Principal Component Analysis

Hierarchical cluster analysis (HCA) and principal component analysis (PCA) are powerful tools for analyzing high-dimensional data sets [13,14,15]. In this study, Cl/Br mass ratios and other groundwater chemicals in various aquifers in the PRD were classified by the HCA using Ward’s method. Relationships between the proportions of contaminated groundwater and socioeconomic parameters in various cities in the PRD were identified by the PCA. Only PCs with eigenvalues > 1 were retained for analyses in PCA, and the varimax method was used.

## 4. Results and Discussion

### 4.1. Identify Groundwater Contamination by the Cl/Br Mass Ratio

#### 4.1.1. Cl/Br Mass Ratios and Hydrochemical Characteristics in Surface Waters and Landfill Leachate in the PRD

**Figure 2:**
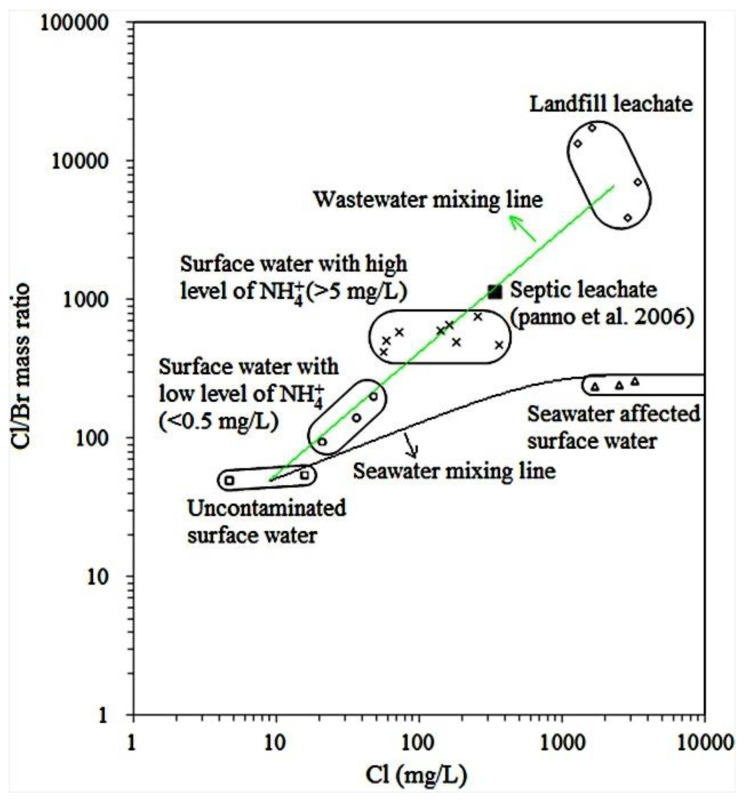
Plot of Cl/Br mass ratios versus Cl concentrations in surface waters in the Pearl River Delta. Also shown are a seawater mixing line between uncontaminated surface water and seawater-affected surface water, a wastewater mixing line linearly connecting uncontaminated surface water, surface water with a low level of NH_4_^+^, surface water with a high level of NH_4_^+^, septic leachate [17], and landfill leachate.

As seen in Table S4, Cl/Br mass ratios in landfill leachates in the PRD were characterized by extremely high values, in the range of 3994 to 17,725. By contrast, Cl/Br mass ratios in drinkable (uncontaminated) surface waters were very low, with a mean value of 53. Cl/Br mass ratios in seawater-affected surface waters ranged from 242 to 265 and were close to that in seawater (Cl/Br mass ratios of 281–292) [16]. Cl/Br mass ratios in river waters in the PRD ranged widely from 96 to 766. It is worth mentioning that low and high values of Cl/Br mass ratios in river waters were accompanied by low and high concentrations of NH_4_^+^, respectively (Figure 2). It is known that NH_4_^+^ is an indicator for surface water contamination in the PRD because sewage in the PRD is commonly accompanied by high concentrations of NH_4_^+^ [5]. Thus, river waters with higher Cl/Br mass ratios and NH_4_^+^ concentrations represent more serious contamination. A wastewater mixing line of the PRD was obtained according to the Cl/Br mass ratios versus Cl concentrations in various waters (Figure 2). In addition, a seawater mixing line of the PRD for Cl/Br mass ratios versus Cl concentrations was also obtained (Figure 2).

#### 4.1.2. Cl/Br Mass Ratios and Hydrochemical Characteristics in Porous Aquifers in the PRD

**Figure 3:**
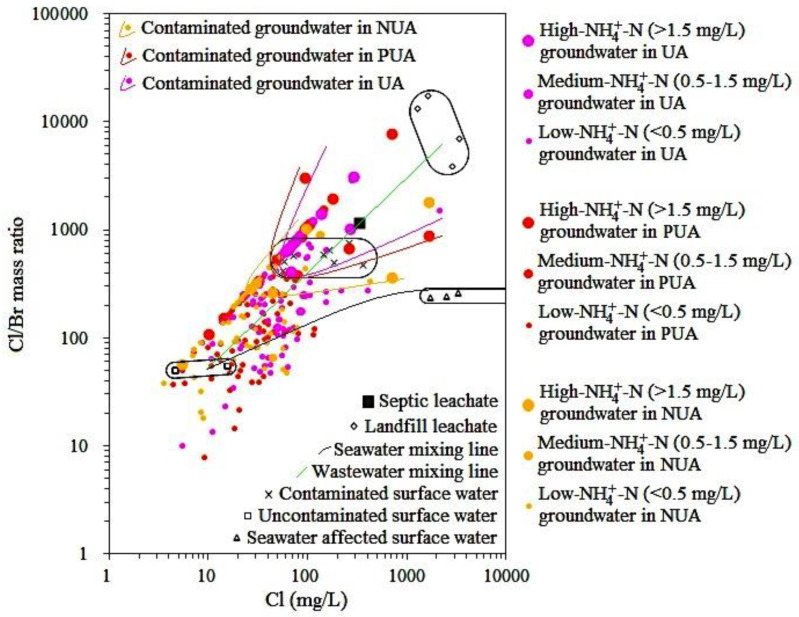
Plot of Cl/Br mass ratios versus Cl concentrations in porous aquifers in areas with different urbanization levels (UA-urbanized area, PUA-peri-urban area, and NUA-non-urbanized area).

In porous aquifers in the PRD, Cl/Br mass ratios ranged from 8 to 7779 (Table S5). The maximum values of Cl/Br mass ratios in porous aquifers at UA, PUA, and NUA were 3148, 7779, and 1841, respectively (Table S5). The mean values of Cl/Br mass ratios in UA and PUA were close to each other and were nearly double that in NUA (Table S5). The relationship between the Cl/Br mass ratio and the concentrations of other components in porous aquifers was investigated by hierarchical cluster analysis (HCA). As seen in Figure S2, the Cl/Br mass ratio and NH_4_^+^ were in the same group and were closer than other components. This indicates that the Cl/Br mass ratio and NH_4_^+^ were effective indicators for groundwater contamination in porous aquifers in the PRD because sewage and contaminated surface water in the PRD were characterized by a high Cl/Br mass ratio and NH_4_^+^ concentration (Figure 2). Similarly, Panno et al. [17] found that contaminated groundwaters, such as septic effluent affected groundwater and animal waste affected groundwater, showed high levels of Cl/Br mass ratios versus Cl concentrations, while groundwater in the pristine aquifer commonly showed low levels of Cl/Br mass ratios versus Cl concentrations. Therefore, in this study, contaminated groundwater in porous aquifers in areas with different urbanization levels was identified by the information of both Cl/Br mass ratios and NH_4_^+^ concentrations. As seen in Figure 3, for contaminated groundwater in porous aquifers in areas with different urbanization levels, three areas with high levels of both Cl/Br mass ratios versus Cl concentrations and NH_4_^+^ concentrations were drawn. Note that some groundwaters with high levels of NH_4_^+^-N (>1.5 mg/L) but not high levels of Cl/Br mass ratios versus Cl concentrations were excluded from these areas because mineralization of organic nitrogen in overlying aquitards is one of the main sources for high levels of NH_4_^+^-N in groundwater in porous aquifers in the PRD [5]. In addition, the urbanization in the PRD accompanied by a relatively anoxic environment led to the higher background value of groundwater NH_4_^+^ in UA than that in NUA [5]. That is why Cl/Br mass ratios versus Cl concentrations in some contaminated groundwater in porous aquifers in NUA were relatively lower than UA (Figure 3).

#### 4.1.3. Cl/Br Mass Ratios and Hydrochemical Characteristics in Fissured Aquifers in the PRD

**Figure 4:**
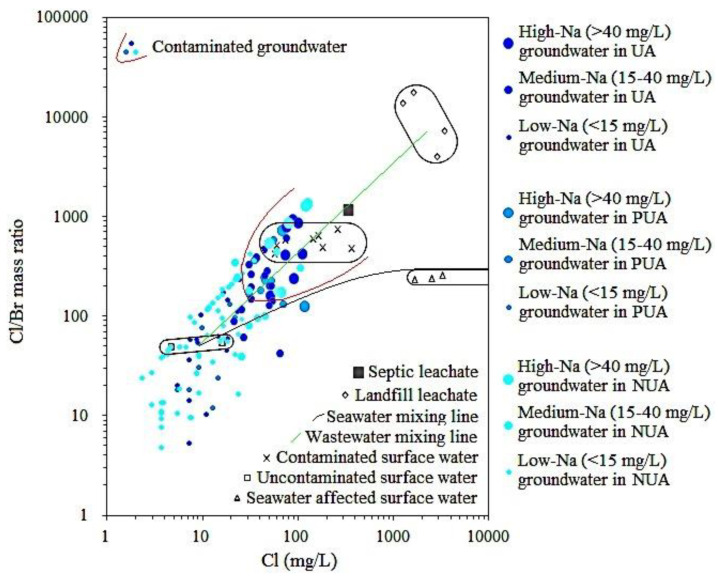
Plot of Cl/Br mass ratios versus Cl concentrations in fissured aquifers in areas with different urbanization levels (UA-urbanized area, PUA-peri-urban area, and NUA-non-urbanized area).

As seen in Table S6, Cl/Br mass ratios in fissured aquifers in the PRD ranged from 5 to 1359. Similar to Cl/Br mass ratios in porous aquifers, their mean values in fissured aquifers in UA and PUA were also close to each other and were much higher than that in NUA. The relationship between the Cl/Br mass ratio and the concentrations of other components in fissured aquifers was also investigated by the HCA. Results showed that the Cl/Br mass ratio and Na^+^ were in the same group (Figure S3). On the other hand, the Cl/Br mass ratio in surface water in the PRD was significantly positively correlated with Na^+^ concentration (Figure S4), and Cl/Br mass ratio and Na^+^ concentration in contaminated river water and sewage such as landfill leachate were much higher than those in drinkable (or uncontaminated) surface water (Table S4). These indicate that the Cl/Br mass ratio and Na^+^ were effective indicators for both groundwater contamination in fissured aquifers and surface water contamination in the PRD. Similarly, Huang et al. [9] also reported that a high concentration of groundwater Na in fissured aquifers was mainly from sewage infiltration. Thus, contaminated groundwater in fissured aquifers was identified by the information of both Cl/Br mass ratios and Na^+^ concentrations. As seen in Figure 4, one area with high levels of both Cl/Br mass ratios versus Cl concentrations and Na^+^ concentrations was circled as contaminated groundwater in fissured aquifers in the PRD. Note that contaminated groundwater in fissured aquifers in areas with different urbanization levels was in one area because of no significant difference in the background value of groundwater Na in fissured aquifers in areas with different urbanization levels [9].

#### 4.1.4. Cl/Br Mass Ratios and Hydrochemical Characteristics in Karst Aquifers in the PRD

**Figure 5:**
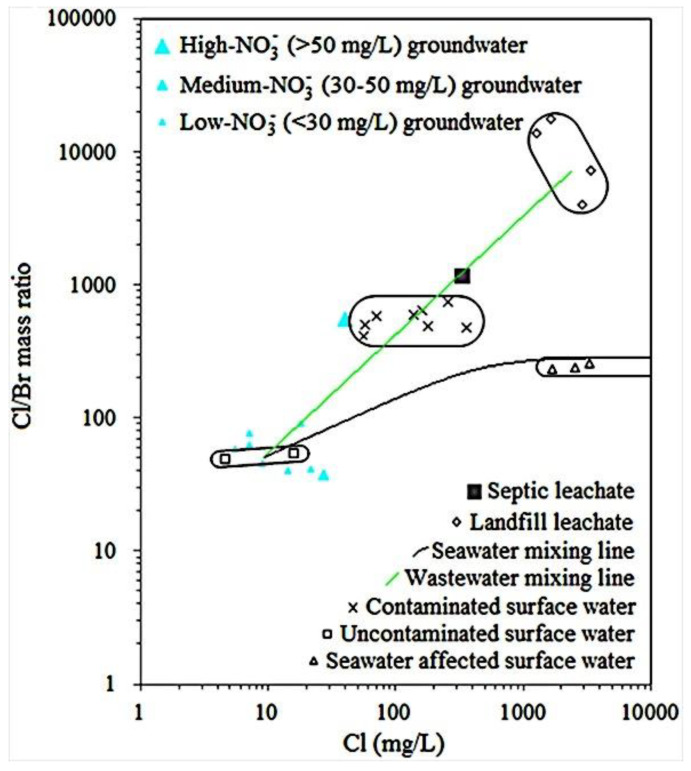
Plot of Cl/Br mass ratios versus Cl concentrations in karst aquifers.

The Cl/Br mass ratio in karst aquifers ranged from 38 to 563, with a mean value of 114 (Table S7). The relationship between the Cl/Br mass ratio and the concentrations of other components in karst aquifers was also investigated by the HCA, and the result showed that the Cl/Br mass ratio and K^+^, NO_3_^−^, and SO_4_^2−^ were in the same group (Figure S5). Similarly, Jiang et al. [18] reported that K^+^, NO_3_^−^, and SO_4_^2−^ are indicators for groundwater contamination in karst aquifers because the main sources of them in karst aquifers are commonly anthropogenic sources, such as sewage effluents and fertilizers. In this study, NO_3_^−^ was chosen to be the indicator for groundwater contamination in karst aquifers because the water-rock interaction is not the source for NO_3_^−^ in karst aquifers but may be the source for K^+^ and SO_4_^2−^ in karst aquifers [9]. Therefore, contaminated groundwater in karst aquifers was identified by the information of both Cl/Br mass ratios and NO_3_^−^ concentrations. As seen in Figure 5, only one sample with high levels (>50 mg/L) of NO_3_^−^ was identified as contaminated groundwater in karst aquifers in the PRD.

### 4.2. Identify Groundwater Contamination by High Levels of NO_3_^−^, Pb, PO_4_^3−^, and Organic Contaminants

In the PRD, high concentrations of NO_3_^−^, PO_4_^3−^, Pb, and organic contaminants in groundwater were attributed to various anthropogenic activities [2,5,10], indicating that these chemicals are useful indicators to identify groundwater contamination in the PRD. Groundwater NO_3_^−^ concentration > 50 mg/L was often used to identify contaminated groundwater samples before the assessment of background levels [6]. Natural background levels of NO_3_^−^ in groundwater are commonly below 50 mg/L [19]. In addition, groundwater samples with NO_3_^−^ concentration > 50 mg/L in the PRD generally occurred in UA and PUA, where human activities are common [5]. Therefore, in this study, NO_3_^−^ concentration > 50 mg/L is used as an indicator for groundwater contamination in the PRD.

Similarly, all groundwater samples with Pb > 0.01 mg/L in the PRD occurred in UA and PUA, where anthropogenic activities are common (Figure S6). By contrast, a large proportion of groundwater samples with lower Pb concentrations (<0.01 mg/L) in the PRD appeared in NUA, where there are fewer anthropogenic activities (Figure S6). In addition, Zhang et al. [2] reported that a high concentration of Pb in groundwater in the PRD was attributed to anthropogenic sources but not geogenic sources. Thus, Pb > 0.01 mg/L is also selected as an indicator for groundwater contamination in the PRD.

As seen in Figure S7, the first significant “gap” from low to high levels of groundwater PO_4_^3−^ in the PRD was around 1.4 mg/L. This indicates that groundwater with PO_4_^3−^ > 1.4 mg/L was probably influenced by human activities because one of our previous publications revealed that a high level of groundwater PO_4_^3−^ in the PRD was mainly attributed to anthropogenic sources [11]. Moreover, almost all groundwater samples with PO_4_^3−^ > 1.5 mg/L in the PRD occurred in UA and PUA, where anthropogenic activities are common; by contrast, groundwater in NUA in the PRD generally showed <0.3 mg/L of PO_4_^3−^ (Figure S8). Thus, PO_4_^3−^ > 1.4 mg/L is also used as an indicator for groundwater contamination in the PRD.

Organic contaminants > 0.05 μg/L are often used as an indicator for groundwater contamination [6], and this value is far lower than the detection limits (0.5–1 μg/L) of organic chemicals in this study. In addition, in the PRD, almost all groundwater samples with detected organic contaminants (>0.5 μg/L) were located at UA and PUA, where human activities are common; by contrast, groundwater samples in NUA far from UA were free of organic contaminants (Figure S9). Thus, organic contaminants > 0.5 μg/L are also used as an indicator for groundwater contamination in this study.

### 4.3. Identify Groundwater Contamination by the Occurrence of NO_3_ Facies

Cl^−^, SO_4_^2−^, HCO_3_^−^, and CO_3_^2−^ are usually major anions in natural groundwater but do not include NO_3_^−^ [20]. This indicates that natural groundwater is free of NO_3_ facies. However, in the PRD, a large amount of groundwater showed NO_3_ facies in UA and PUA after three decades of urbanization (Figure S10). In addition, Huang et al. [9] reported that large-scale urbanization is the main driving force for the occurrence of NO_3_ facies in groundwater in the PRD and that groundwater in the PRD was free of NO_3_ facies before large-scale urbanization (GHST, 1981). Therefore, the occurrence of NO_3_ facies is also used as an indicator for groundwater contamination in this study.

### 4.4. Spatial Distribution of Contaminated Groundwater in the PRD

**Figure 6:**
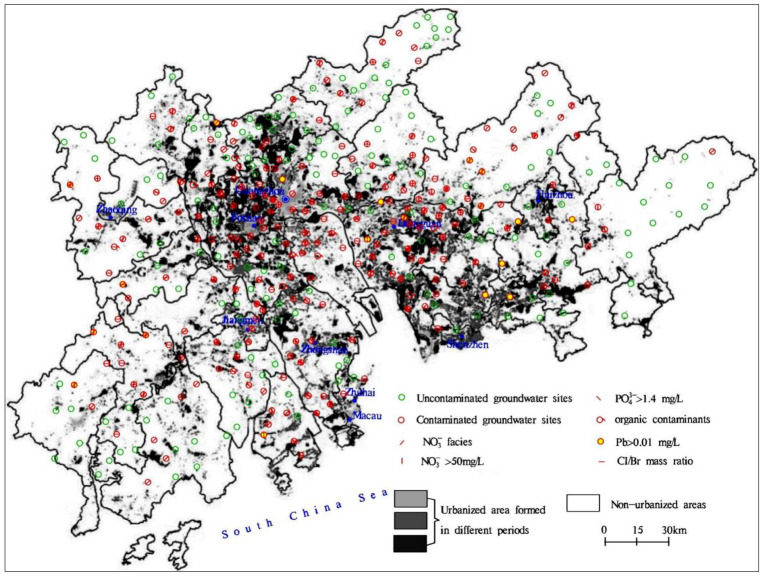
Spatial distribution of groundwater contamination in the Pearl River Delta.

As seen in Figure 6, nearly 60% of groundwater in the PRD was identified to be contaminated according to the previously mentioned 6 indicators. The proportions of contaminated groundwater in porous and fissured aquifers were more than 60% and 50%, respectively, and both of them were more than 2 times that in karst aquifers (Figure 6). This indicates that porous and fissured aquifers have a higher risk of groundwater contamination than karst aquifers in the PRD, and the groundwaters in porous and fissured aquifers are easier to contaminate in comparison with that in karst aquifers. This is probably attributed to the lower groundwater vulnerability of karst aquifers with surface cover layers in comparison with porous and fissured aquifers [21].

In porous aquifers, the proportion of contaminated groundwater in UA was the highest and was approximately 1.2 times and 1.4 times those in PUA and NUA, respectively (Figure 6). This indicates that groundwater contamination in porous aquifers is positively correlated with the urbanization level. By contrast, in fissured aquifers, the proportions of contaminated groundwater in UA and PUA were more than 70%, and both were about 2 times that in NUA (Figure 6). This indicates that higher urbanization levels also result in a higher risk of groundwater contamination in fissured aquifers.

As shown in Table S8, the proportion of contaminated groundwater in Dongguan was 80%, and it was the highest in the PRD. Followed by Zhuhai, Foshan, and Zhongshan, and the proportions of contaminated groundwater in these three cities ranged from 60–70%. Jiangmen, Huizhou, Guangzhou, and Zhaoqing had a range of 50–60% for proportions of contaminated groundwater. The proportion of contaminated groundwater in Shenzhen was the lowest at <50%.

### 4.5. Factors Controlling Groundwater Contamination in the PRD

Anthropogenic factors resulting in groundwater contamination are often quantified by socioeconomic parameters [22,23]. Thus, in order to understand which anthropogenic factors are mainly responsible for groundwater contamination in the PRD, the relationship between the contaminated groundwater and socioeconomic parameters in various cities in the PRD was investigated by the PCA. As seen in Table 1, the PC2 had strong positive loadings (>0.75) with the amount of township-village enterprises, proportions of contaminated groundwater, and the amount of industrial wastewater discharge in nine major cities in the PRD. This indicates that wastewater from township-village enterprises was likely to be the main source for groundwater contamination in the PRD, because wastewater from township-village enterprises was often discharged directly into the nearby ground surface without treatment to reduce the production cost in the PRD before 2006 [1,5]. For example, the amounts of township-village enterprises and industrial wastewater discharge in Dongguan were higher than those in other cities (except Zhongshan) in 2006 (Table S3), and the proportion of contaminated groundwater in Dongguan was the highest (Table S8). By contrast, Shenzhen had the lowest amount of township-village enterprises in 2006 (Table S3). Similarly, Zhaoqing had the lowest amount of industrial wastewater discharge in 2006 (Table S3), and the proportions of contaminated groundwater in these two cities were lower than that in other cities (Table S8). Therefore, the infiltration of wastewater from township-village enterprises was mainly responsible for the occurrence of groundwater contamination in the PRD on a regional scale. Note that the PC3 had weak negative loadings (<−0.3) with the proportions of contaminated groundwater and the values of livestock density in nine major cities in the PRD (Table 1). This indicates that livestock waste was not the main source but was an important source for groundwater contamination in the PRD because livestock waste was an important factor resulting in groundwater PO_4_^3−^ and NO_3_^−^ contamination [5,11].

## 5. Conclusions

The contaminated groundwater in the PRD was identified by six indicators, including the Cl/Br ratio, the NO_3_ facies, the concentrations of NO_3_^−^, PO_4_^3−^, organic contaminants, and Pb in groundwater. Note that the Cl/Br ratio was combined with high levels of NH_4_^+^, Na, and NO_3_^−^ for identifying contaminated groundwater in porous aquifers, fissured aquifers, and karst aquifers, respectively. Moreover, the criteria of NO_3_^−^ and Pb concentrations used for distinguishing groundwater contamination are within the allowable limits for drinking purposes recommended by the World Health Organization [24]. Our results showed that approximately 60% of groundwater sites in the PRD were contaminated. The proportions of contaminated groundwater in porous and fissured aquifers were more than 2 times that in karst aquifers. Groundwater contamination in porous aquifers is positively correlated with the urbanization level. Similarly, in fissured aquifers, the proportions of contaminated groundwater in urbanized and peri-urban areas were approximately 2 times that in non-urbanized areas.

Results from the relationship between the contaminated groundwater and socioeconomic parameters indicate that groundwater contamination in the PRD was mainly attributed to the infiltration of wastewater from township-village enterprises on a regional scale. In addition, livestock waste was also an important source of groundwater contamination in the PRD. Therefore, in the future, the supervision of the wastewater discharge of township-village enterprises and the waste discharge of livestock should be strengthened to protect against groundwater contamination in the PRD.

## Figures and Tables

**Table 1:** Principal component (PC) loadings for groundwater contamination and socioeconomic parameters in nine major cities in the Pearl River Delta.

Items	PCs		
	PC1	PC2	PC3
GDP	***0.993***	−0.115	−0.009
PD	***0.985***	0.133	−0.038
DSD	***0.982***	0.035	0.149
UR	***0.940***	0.098	−0.209
IE	***0.850***	0.393	−0.089
TVE	−0.016	***0.894***	0.343
PCG	−0.039	***0.870***	−0.351
IWD	0.412	***0.859***	−0.125
LO	−0.115	−0.267	***0.918***
AO	−0.268	−0.001	***0.899***
LD	−0.229	−0.099	−0.412
Eigenvalue	4.8	2.6	2.2
Explained variance (%)	44.0	23.4	19.6
Cumulative % of variance	44.0	67.3	86.9

Bold and italic numbers: maximum absolute PC loading for one parameter. GDP: gross domestic product; PD: population density; DSD: domestic sewage discharge; UR: urbanization ratio; IE: industrial enterprises above designated size; TVE: township-village enterprises; PCG: proportions of contaminated groundwater. IWD: industrial wastewater discharge; LO: livestock output; AO: agricultural output; LD: livestock density.

## Data Availability

The datasets generated and/or analyzed during the current study are not publicly available.

## Supplementary Materials

The following are available online at https://www.mdpi.com/article/10.3390/ijerph182212143/s1, Figure S1: Urban expansion and sampling sites in the PRD, Figure S2: Relationship of Cl/Br mass ratio and other components in porous aquifers in the PRD, Figure S3: Relationship of Cl/Br mass ratio and other components in fissured aquifers in the PRD, Figure S4: Relationship of Cl/Br mass ratio and Na concentration in surface water (excludes 3 estuary water) in the PRD, Figure S5: Relationship of Cl/Br mass ratio and other components in karst aquifers in the PRD, Figure S6: Spatial distribution of groundwater Pb concentration in the PRD (data from Zhang et al., [25]). Figure S7: Statistics for groundwater PO43– concentrations in the PRD, Figure S8: Spatial distribution of groundwater PO43– concentration in the PRD (data from Huang et al., [26]), Figure S9: Spatial distribution of groundwater organic contaminants in the PRD (data from Huang et al., [27]), Figure S10: Spatial distribution of hydrochemical facies for groundwater in the PRD (data from Huang et al., [28]), Table S1: Detection limits of inorganic parameters in water, Table S2: 55 selected organic chemicals in the groundwater of the Pearl River Delta (PRD), Table S3: Socioeconomic data for the nine major cities of the PRD in 2006, Table S4: Statistics of concentrations of chemical components and Cl/Br mass ratios in surface waters and landfill leachate in the PRD, Table S5: Statistics of concentrations of chemical components and Cl/Br mass ratios in porous aquifers in the PRD, Table S6: Statistics of concentrations of chemical components and Cl/Br mass ratios in fissured aquifers in the PRD, Table S7: Statistics of concentrations of chemical components and Cl/Br mass ratios in karst aquifers in the PRD, Table S8: Proportions of contaminated groundwater in various cities in the PRD.
