# Supplementary material for: Identification of Groundwater Contamination in a Rapidly Urbanized Area on a Regional Scale: A New Approach of Multi-Hydrochemical Evidences
Source: Int J Environ Res Public Health. 2021 Nov 19;18(22):12143. doi: 10.3390/ijerph182212143 (PMC8623665; doi:10.3390/ijerph182212143)
Supplement: Supplementary file 1 [file ijerph-18-12143-s001.zip › ijerph-1455491-supplementary.pdf]

Article

Identification of the groundwater contamination in a rapidly urbanized area on a regional scale: A new approach of multi-hydrochemical evidences

Pan Bi [1,2], Lixin Pei [3,*], Guanxing Huang [4,*], Dongya Han [4], Jiangmin Song [4]

[1] School of Water Resources and Environment, Hebei GEO University, Shijiazhuang, China
[2] Hebei Province Collaborative Innovation Center for Sustainable Utilization of Water Resources and Optimization of Industrial Structure, Hebei GEO University, China
[3] Haikou Marine Geological Survey Center, China Geological Survey, Haikou, China
[4] Institute of Hydrogeology and Environmental Geology, Chinese Academy of Geological Sciences, Shijiazhuang, China
* Correspondence: author E-mail address: 107826726@qq.com (L. Pei); huangguanxing@mail.cgs.gov.cn (G. Huang)

Table S1. Detection limits of inorganic parameters in water.

Chemical parameters	Detection limits (mg/L)
TDS	4
COD	0.5
K^+	0.1
Na^+	1
Ca^{2+}	3
Mg^{2+}	3
HCO_3^-	3
Cl^-	2
SO_4^{2-}	2
NO_3^-	0.2
NH_4^+	0.02
Pb	0.002
PO_4^{3-}	0.01
Br^-	0.1

Table S2. 55 selected organic chemicals in the groundwater of the Pearl River Delta (PRD).

Polycyclic aromatic hydrocarbons (PAHs) (16)	Chlorobenzenes (CBs) (8)	Monocylic aromatics (MAs) (10)	Halogenated aliphatic hydrocarbons (HAHs) (21)
Naphthalene	Chlorobenzene	Benzene	1,2-Dichloropropane
2-Methylnaphthalene	1,3-Dichlorobenzene	Toluene	Hexachloroethane
Acenaphthylene	1,4-Dichlorobenzene	Ethylbenzene	1,1-Dichloroethane
Acenaphthene	1,2-Dichlorobenzene	m-,p-Xylene	1,1,1-Trichloroethane
Fluorene	1,2,4-Trichlorobenzene	Styrene	1,2-Dibromoethane
Phenanthrene	1,2,3-Trichlorobenzene	o-Xylene	1,2-Dichloroethane
Anthracene	Pentachlorobenzene	Isopropylbenzene	1,1,2-Trichloroethane
Fluoranthene	Hexachlorobenzene	n-Propylbenzene	1,1,1,2-Tetrachloroethane
Pyrene		1,3,5-Trimethylbenzene	1,1,2,2-Tetrachloroethane
Benzo(a)anthracene		1,2,4-Trimethylbenzene	Chloroform
Chrysene			Bromodichloromethane
Benzo(bk)fluoranthene			Dibromochloromethane
Benzo(a)pyrene			Bromoform
Indeno(123-cd)pyrene			Carbon tetrachloride
Dibenzo(ah)anthracene			Dibromomethane
Benzo(ghi)perylene			Tetrachloroethylene
			Trichloroethylene
			cis-1,2-Dichloroethylene
			trans-1,2-Dichloroethylene
			1,1-Dichloroethylene
			Vinyl chloride

Table S3. Socioeconomic data for the nine major cities of the PRD in 2006.

Socioeconomic parameters	Guangzhou	Shenzhen	Foshan	Dongguan	Huizhou	Zhaoqing	Jiangmen	Zhuhai	Zhongshan
PD (people/km^2)	1312	4334	1522	2738	337	249	431.00	859	1385
GDP (Chinese million Yuan/km^2)	81.7	297.7	76.1	106.6	8.4	3.5	9.90	44.3	57.6
DSD (tons/km^2)	14.51	34.49	6.61	18.96	1.38	0.50	1.54	7.03	8.78
IWD (tons/km^2)	2.75	3.27	5.84	9.79	0.56	0.50	1.27	2.03	5.71
UR (%)	19.62	66.27	32.47	36.78	3.30	3.99	7.10	33.86	24.04
IE (number/km^2)	0.70	2.63	1.56	1.86	0.11	0.05	0.26	0.58	2.07
TVE (number/km^2)	9.55	0.00	1.69	34.15	7.34	3.91	9.99	5.28	37.16
AO (Chinese million Yuan/km^2)	1.67	0.20	0.17	0.43	0.60	0.66	0.56	0.39	0.96
LO (Chinese million Yuan/km^2)	0.74	0.34	0.13	0.23	0.28	0.43	0.45	0.30	0.44
LD (number/km^2)	463	311	891	211	317	457	433	375	493

PD: population density; GDP: gross domestic product; DSD: domestic sewage discharge; IWD: industrial wastewater discharge; UR: urbanization ratio; IE: industrial enterprises above designated size; TVE: township–village enterprises; AO: agricultural output; LO: livestock output; LD: livestock density.

Table S4. Statistics of concentrations of chemical components and Cl/Br mass ratios in surface waters and landfill leachate in the PRD.

Surface water and leachate	Values	K^+	Na^+	Ca^{2+}	Mg^{2+}	Br^-	HCO_3^-	Cl^-	SO_4^{2-}	NO_3^-	TDS	PO_4^{3-}	Pb	NH_4^+	COD	Cl/Br mass ratios
		mg/L														
Landfill leachate	Min	113.5	139	9.6	93.8	–	2837	1245	–	22.3	6279	2.04	0.006	150	154	3994
	Max	1624.8	2026	771	156	0.71	12915	3334	12.6	272.1	25662	29.28	0.073	2800	2843	17725
	Mean	855.9	1135	322.9	128.4	0.33	6909	2253	4.5	130.7	13590	21.82	0.039	1193	1288	10699
River water	Min	4.1	14.7	21.1	2.9	0.11	25.5	20.2	11.3	0.5	328	0.16	–	0.11	2.15	96
	Max	50	161.3	128.7	27.5	0.73	679.7	354.7	107.5	47.4	1660	10.08	0.581	42	21.68	766
	Mean	17.1	76.6	45.6	9.5	0.27	205.3	124.4	58.1	11.9	627	4.7	0.057	15.36	11.08	455
Seawater-affected surface water (estuaries water)	Min	108.4	871	52.7	109.8	6.81	81.8	1648	196	14.7	3101	0.25	0.002	0.2	6.99	242
	Max	133.2	1537	82.7	200.8	12.05	103.8	3191	368	18.2	5643	0.58	0.004	2.8	11.65	265
	Mean	122.4	1245	67.5	162.2	9.6	91.2	2434	306	16.1	4458	0.36	0.003	1.1	9.84	252
Drinkable/uncontaminated surface water	Min	0.7	1.5	5.6	0.3	–	13.9	4.5	5.3	2.50	40.1	0.06	0.001	0.2	0.12	50
	Max	3	9	12.4	1.7	0.28	25.5	15.50	14.90	4.1	99.7	0.19	0.005	0.4	2.45	55
	Mean	1.8	5.2	9	1	0.14	19.7	10	10.1	3.3	69.9	0.13	0.003	0.3	1.29	53

–: below detection limits.

Table S5. Statistics of concentrations of chemical components and Cl/Br mass ratios in porous aquifers in the PRD.

**Areas with different urbanization levels**	**Values**	**K^+**	**Na^+**	**Ca^{2+}**	**Mg^{2+}**	**Br^-**	**HCO_3^-**	**Cl^-**	**SO_4^{2-}**	**NO_3^-**	**TDS**	**PO_4^{3-}**	**Pb**	**NH_4^+**	COD	Cl/Br mass ratios
		**mg/L**														
Urbanized areas	min	1.1	1.7	4.4	–	–	3	5.3	–	0.3	79	–	–	–	0.54	10
	max	119.9	954.7	262.5	93.6	1.38	748.1	2042.8	324.3	333.5	3734	9.86	0.043	30	26.88	3148
	mean	25.4	51.7	66.9	9.6	0.25	198	82.6	48.1	40.4	545	1.23	0.003	1.3	2.77	425
Peri-urban areas	min	0.4	1.5	2.4	–	–	3	4.3	–	–	45	–	–	–	–	8
	max	102.3	1008.8	141.6	93.6	1.31	616.3	1630.7	210.8	146.8	3353	7.86	0.05	60	33.81	7779
	mean	19.2	47.9	52.1	8.1	0.25	163.9	71.2	39.5	30.2	452	0.77	0.003	2.22	2.85	420
Non-urbanized areas	min	1	1.6	2	–	–	3	3.5	–	0.8	29	–	–	–	–	18
	max	56.5	802.5	98.5	118.5	1.85	459	1620	126.6	116.4	3152	4.13	0.015	40	10	1841
	mean	16.6	38.6	31.5	8.6	0.25	100.8	70.6	24.4	22.9	325	0.26	0.002	1.07	1.6	230

–: below detection limits.

Table S6 Statistics of concentrations of chemical components and Cl/Br mass ratios in fissured aquifers in the PRD

**Areas with different urbanization levels**	**Values**	**K^+**	**Na^+**	**Ca^{2+}**	**Mg^{2+}**	**Br^-**	**HCO_3^-**	**Cl^-**	**SO_4^{2-}**	**NO_3^-**	**TDS**	**PO_4^{3-}**	**Pb**	**NH_4^+**	COD	Cl/Br mass ratios
		**mg/L**														
**Urbanized areas**	**min**	**0.6**	**2.5**	**3.2**	**–**	**–**	**4.2**	**5.3**	**–**	**1.6**	**45**	**–**	**–**	**–**	–	5
	max	73.1	85.5	114.8	42.2	1.47	426.8	108.6	78.2	153.6	864	8.36	0.023	2.2	5.08	955
	mean	15.5	25.2	27.4	4.8	0.27	76.4	37	18	38.4	257	0.54	0.003	0.21	1.56	227
Peri-urban areas	min	0.3	3	3.6	–	–	3	5.3	–	0.7	36	–	–	–	–	12
	max	116.8	55.2	101.8	9.1	1.01	374.5	112.3	132.4	153.9	832	2.64	0.016	0.96	5.13	733
	mean	24	21.4	30.5	4.7	0.35	85.2	35.2	26.4	39.1	281	0.26	0.002	0.1	1.58	202
Non-urbanized areas	min	0.4	–	2	–	–	3	2.2	–	–	17	–	–	–	0.5	5
	max	56.2	76.5	99.2	12.6	1.35	225.3	122.3	81.9	145.4	553	3.35	0.046	2.4	9.17	1359
	mean	9.2	12.3	19	2.5	0.2	50.6	20.6	8.8	23.3	161	0.11	0.004	0.09	1.32	154

–: below detection limits.

Table S7. Statistics of concentrations of chemical components and Cl/Br mass ratios in karst aquifers in the PRD.

**Values**	**K^+**	**Na^+**	**Ca^{2+}**	**Mg^{2+}**	**Br^-**	**HCO_3^-**	**Cl^-**	**SO_4^{2-}**	**NO_3^-**	**TDS**	**PO_4^{3-}**	**Pb**	**NH_4^+**	COD	Cl/Br mass ratios
	**mg/L**														
min	0.2	1.8	5.1	–	–	10.9	5.3	–	1.8	60	–	–	–	–	38
max	30	16.8	42.3	8.4	0.71	155.6	39.4	71.5	53.5	299	0.6	0.029	0.1	2.48	563
mean	10	9.1	23.9	3.7	0.25	59.3	16.3	22.3	21.7	174	0.15	0.004	0.04	1.21	114

–: below detection limits.

Table S8. Proportions of contaminated groundwater in various cities in the PRD.

**Item**	**Guangzhou**	**Shenzhen**	**Foshan**	**Dongguan**	**Huizhou**	**Zhaoqing**	**Jiangmen**	**Zhuhai**	**Zhongshan**
Proportions of contaminated groundwater (%)	50.5	46.7	66.1	80.0	51.6	50.0	55.6	69.2	63.2

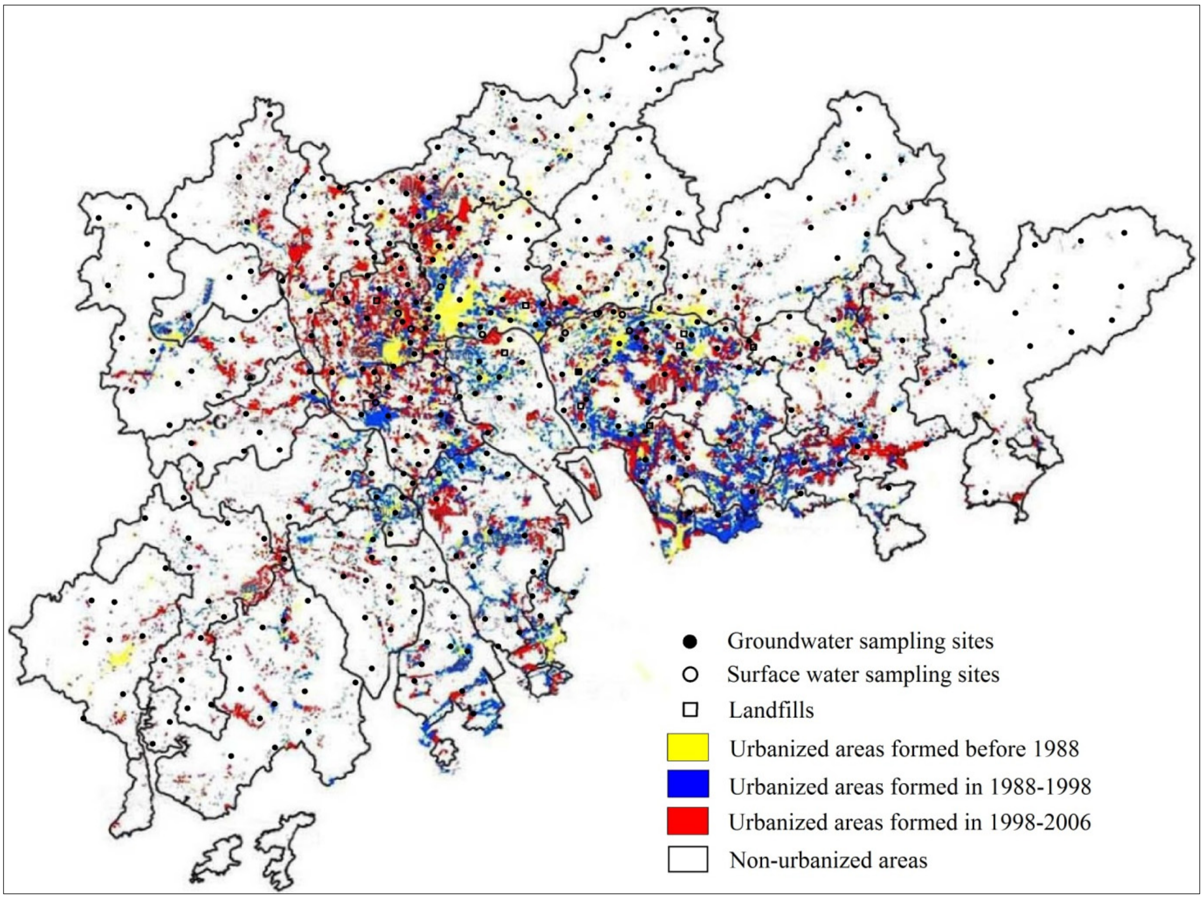

Figure S1. Urban expansion and sampling sites in the PRD.

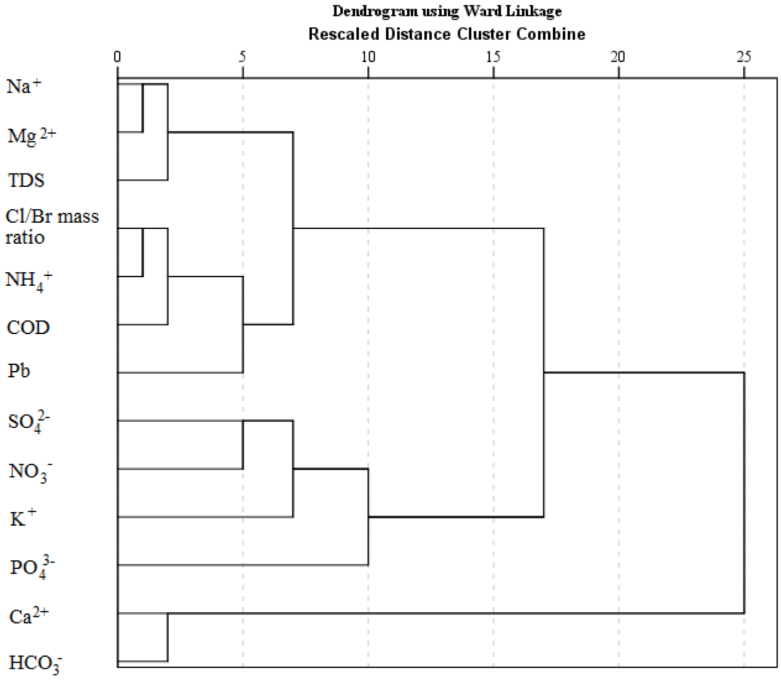

Figure S2. Relationship of Cl/Br mass ratio and other components in porous aquifers in the PRD.

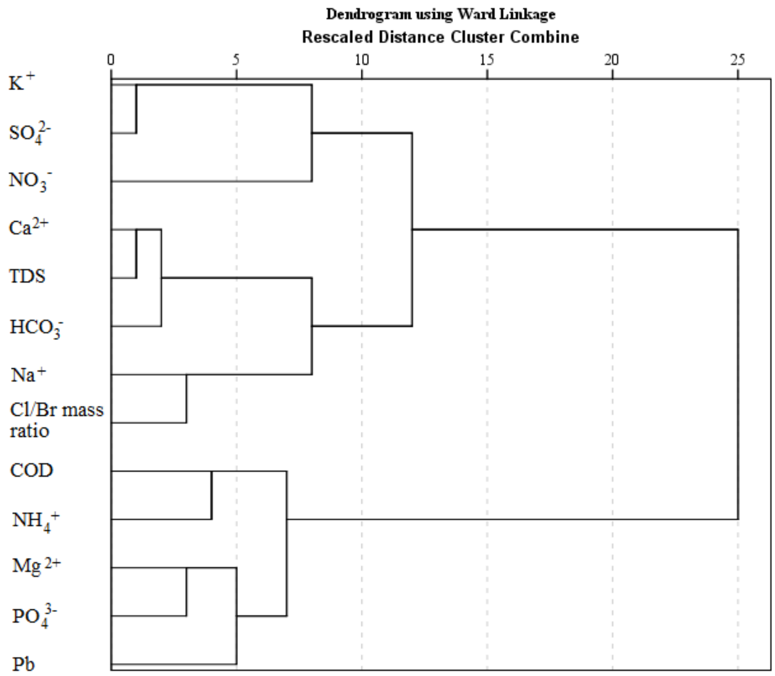

Figure S3. Relationship of Cl/Br mass ratio and other components in fissured aquifers in the PRD.

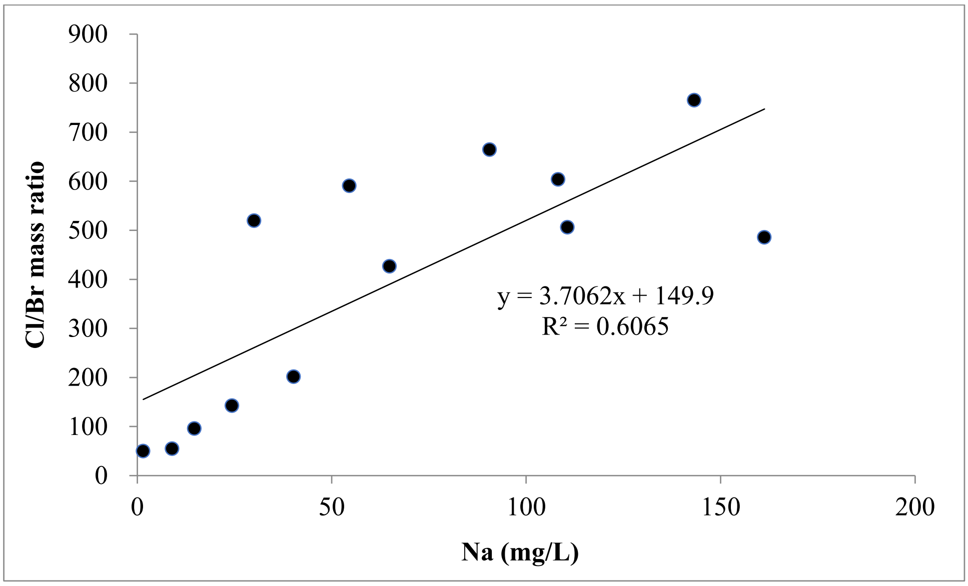

Figure S4. Relationship of Cl/Br mass ratio and Na concentration in surface water (excludes 3 estuary water) in the PRD.

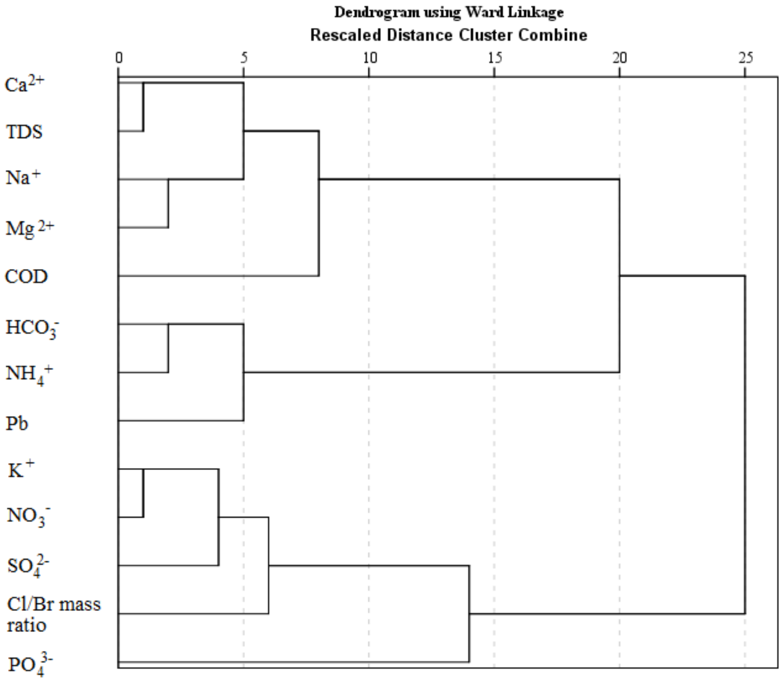

Figure S5. Relationship of Cl/Br mass ratio and other components in karst aquifers in the PRD.

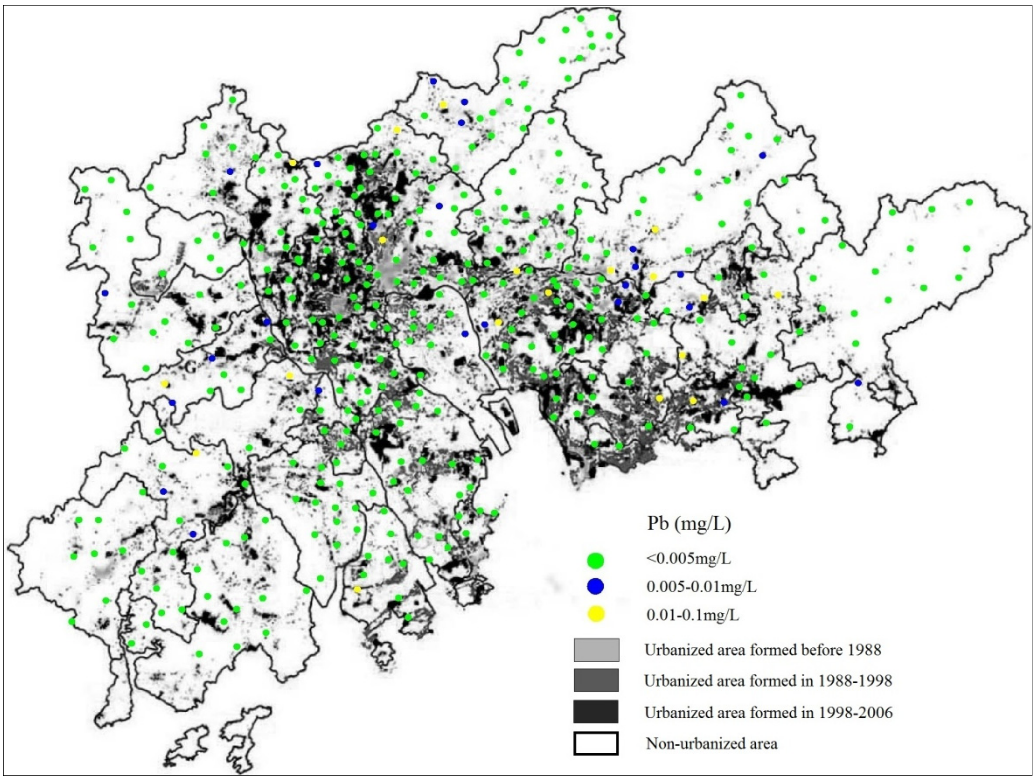

Figure S6. Spatial distribution of groundwater Pb concentration in the PRD (data from Zhang et al. (2019)).

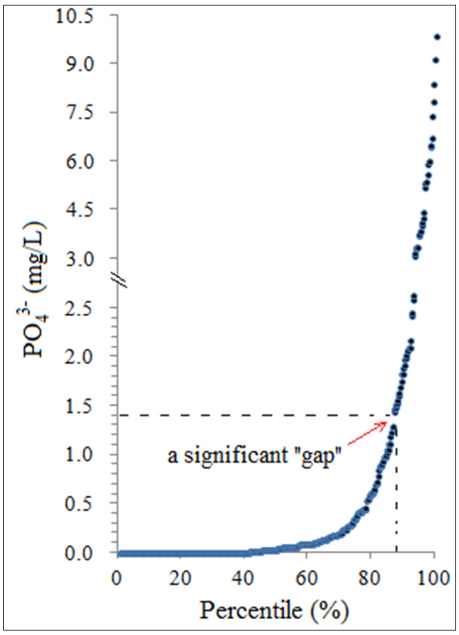

Figure S7. Statistics for groundwater PO_4^{3-} concentrations in the PRD.

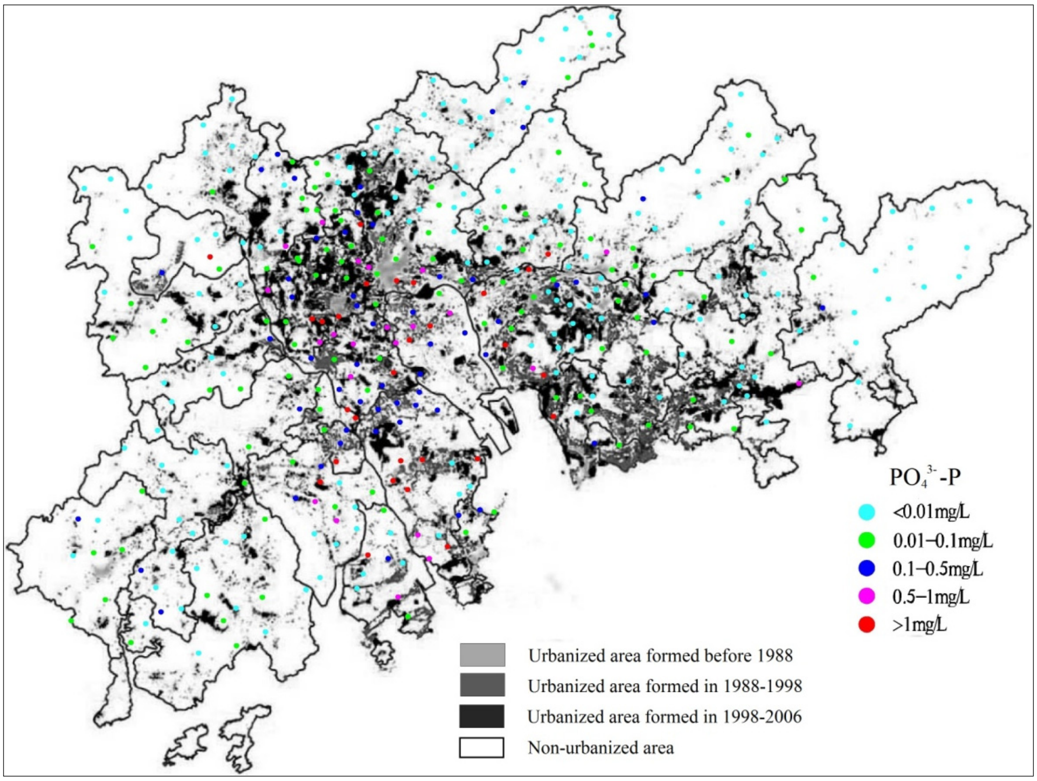

Figure S8. Spatial distribution of groundwater PO_4^{3-} concentration in the PRD (data from Huang et al. (2020)).

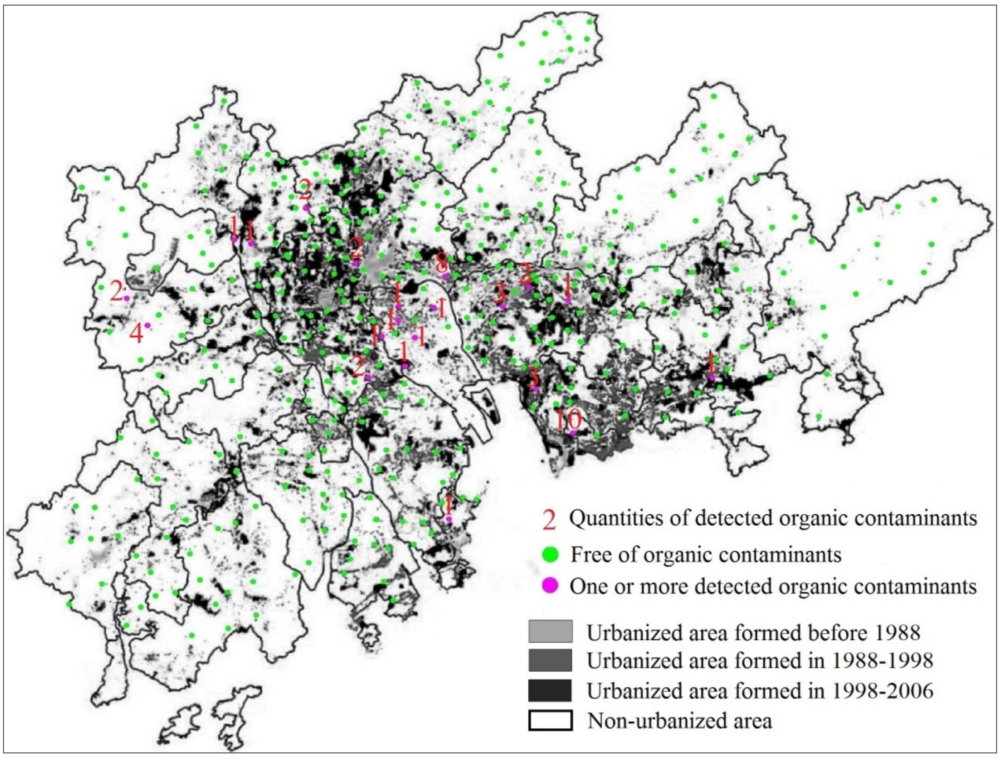

Figure S9. Spatial distribution of groundwater organic contaminants in the PRD (data from Huang et al. (2018b)).

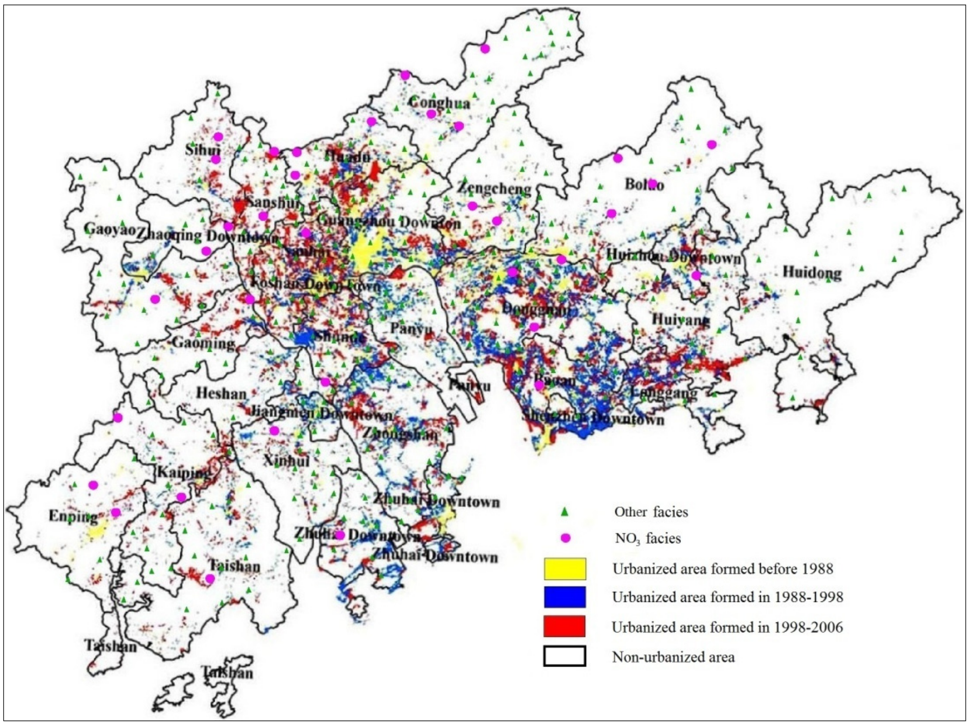

Figure S10. Spatial distribution of hydrochemical facies for groundwater in the PRD (data from Huang et al. (2018a)).

References

25. Zhang, F.; Huang, G.; Hou, Q.; Liu, C.; Zhang, Y.; Zhang, Q. Groundwater quality in the Pearl River Delta after the rapid expansion of industrialization and urbanization: Distributions, main impact indicators, and driving forces. *J. Hydrol.* **2019**, *577*, 124004. https://doi.org/10.1016/j.jhydrol.2019.124004.
26. Huang, G.; Liu, C.; Zhang, Y.; Chen, Z. Groundwater is important for the geochemical cycling of phosphorus in rapidly urbanized areas: A case study in the Pearl River Delta. *Environ. Pollut.* **2020**, *260*, 114079. https://doi.org/10.1016/j.envpol.2020.114079.
27. Huang, G.; Zhang, M.; Liu, C.; Li, L.; Chen, Z. Heavy metal(loid)s and organic contaminants in groundwater in the Pearl River Delta that has undergone three decades of urbanization and industrialization: Distributions, sources, and driving forces. *Sci. Total Environ.* **2018**, *635*, 913–925.
28. Huang, G.; Liu, C.; Sun, J.; Zhang, M.; Jing, J.; Li, L. A regional scale investigation on factors controlling the groundwater chemistry of various aquifers in a rapidly urbanized area: A case study of the Pearl River Delta. *Sci. Total Environ.* **2018**, *625*, 510–518.
